# The Hybrid Fabrication Process of Metal/Silicon Composite Structure for MEMS S&A Device

**DOI:** 10.3390/mi10070469

**Published:** 2019-07-13

**Authors:** Tengjiang Hu, Kuang Fang, Zhiming Zhang, Xiaohua Jiang, Yulong Zhao

**Affiliations:** 1 State Key Laboratory for Manufacturing System Engineering, Xi’an Jiaotong University, Xi’an 710049, China; 2 Institute of Chemical Materials, China Academy of Engineering Physics, Mianyang 621900, China

**Keywords:** hybrid fabrication process, metal/silicon composite structure, micro-electromechanical-system (MEMS) safety-and-arming (S&A) device

## Abstract

The micro-electromechanical system (MEMS) safety-and-arming (S&A) device has the features of integration and miniaturization, which is one of the important directions of weapon development. Confined by the fabrication process, the silicon-based devices are too fragile, and the metal-based devices are low precision. In order to solve the contradiction between high precision and high structure strength, a metal/silicon composite structure is proposed in this paper, and a hybrid fabrication process is introduced. This new method mainly consists of metal sputtering, electroplating, and (inductively–coupled-plasma) ICP etching. As the resolution of the thick dry film is limited, the process of a femtosecond laser is applied to refine the structure, and the Ni plate (a block of 1 mm × 3 mm × 0.3 mm with a cavity of ϕ 0.85 mm × 0.3 mm in the center) is fabricated on the silicon-on-insulator (SOI) wafer successfully. After the double sides are etched by ICP, the SOI wafer is immersed in a buffered-oxide-etch (BOE) etchant to remove the buried layer. The cover plate acts as the encapsulation and is bonded with the SOI wafer by the epoxy glue. Then, the temporary support beam of the device is broken by the probe, and the suspended composite structure can be fully released. The hybrid process is the integration of the silicon-based process and the metal-based process, which can combine the advantages of both high precision and a high structure strength. The process proposed here is suitable for the application of weapon miniaturization.

## 1. Introduction

Safety-and-arming (S&A) devices are one of the core components in munitions, and are mainly applied in the micro initiator. As the most sensitive part in the weapon system, its great damage ability makes it necessary to design the micro-electromechanical system (MEMS) S&A device, with which the function of energy control can be realized. Confined by the fabrication method, the present S&A device can hardly meet the demands of the minimized size and high structure strength [1,2].

Micro-electromechanical-system (MEMS) technology has been explored in this field recently. According to the different fabrication methods, the MEMS S&A device can be divided into two categories—a metal-based device [3,4,5,6,7,8] and a silicon-based device [9,10,11,12]. Ni is commonly used in the metal-based device. The micro structures, such as sliders, springs, and latches, are fabricated by lithographie-galvanoformung-abformung (LIGA) separately, and then are assembled together. As a result of the high strength material property, the device can stay intact under a high impact, which can greatly improve the safety of the weapon system [7,8]. Unfortunately, the LIGA process is costly, and confined by the low resolution of the thick photoresist, the fabrication precision is not satisfactory [13]. In the silicon-based device, the structure is usually achieved by micromachining, which gives the device the features of high-precision and -integration [14,15,16]. While, confined by the fragility of the silicon material, the silicon structure will break into pieces when under a high-impact. Although the energetic material cannot be triggered, it still poses a safety hazard [11]. Helene Pezous et al. [1] have found a special way to combine the advantage of a metal material and silicon material. In their work, they have separated the device into the logic control part and the barrier part. The logic control part is fabricated on a silicon wafer, and the barrier part is based on aluminum. Then, these two parts are glued together to achieve the device assembly. The ultimate size of the device is less than 10 mm × 10 mm × 10 mm. Nonetheless, because of the manual assembly, the fabrication quality and efficiency are limited. Feng [17] has reported the integration of a MEMS S&A device with different films (silver, copper, nickel, and polyimide), and the experiment results show that the performance of the explosion suppression can be improved greatly. However, the fabrication method proposed by Feng is simply electroplating or coating another film on the silicon structure. Without patterning, all of the structures are covered by the enhanced film, which may cause damage to the already formed structure. In summary, the metal-based device has the unique advantage of being explosion-proof, while the device is less intelligent and hard to be minimized. On the contrary, the silicon-based device can achieve fine structures and complex functions. The shortcoming of the silicon device is that it is too fragile to be applied in a weapon system.

In order to solve the contradiction above, we present a MEMS S&A device with a hybrid structure, which can combine the advantages of the metal device and silicon device together. In the fabrication flow, a 300-µm thick Ni plate has been electroplated and patterned on the silicon-on-insulator (SOI) wafer. In order to avoid the structure distortion caused by the low resolution of the thick photoresist, a laser process has been applied to refine the structure. After being etched by (inductively–coupled-plasma) ICP on both sides, the SOI wafer then forms a suspend metal/silicon composite structure. The cover plate is introduced here as the encapsulation, and is fabricated on another silicon wafer. Epoxy glue (EPO 330) has been used here to accomplish bonding between the SOI wafer and the cover plate wafer. After dicing by laser, the ultimate size of the device is 10.5 mm × 14.2 mm × 0.75 mm. The device fabricated by the hybrid process can have features such as a minimized size, high precision, and high structure strength, which is conducive to the development of weapon miniaturization.

## 2. The Hybrid Process

### 2.1. The Basic Structure of a MEMS S&A Device

**Figure 1:**
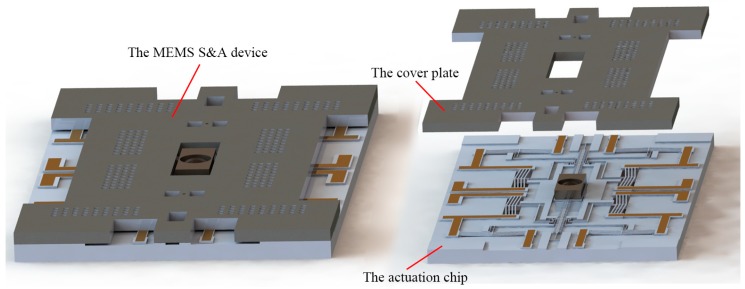
The basic structure of micro-electromechanical-system (MEMS) safety-and-arming (S&A) device.

Different from the traditional macro process, the fabrication sequence of the MEMS device is not standard, and can be affected by the structure size and shape drastically. In order to find out the proper process flow, the basic structure of the MEMS S&A device is introduced in this section, as shown in Figure 1. The device is composed of a cover plate and an actuation chip. The cover plate acts as the encapsulation, and protects the whole chip from the hostile environment.

**Figure 2:**
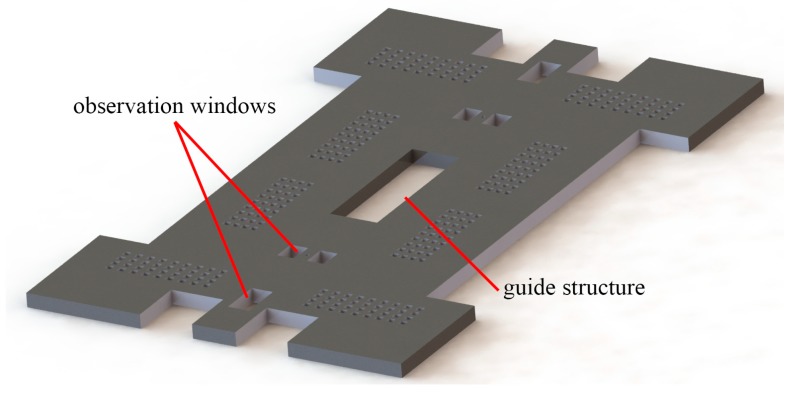
The structure of the cover plate.

The structure of the cover plate is shown in Figure 2, and the main functions are as follows: (1) Constrain the out-off-plane movement of the device. (2) Observe every step movement of the actuation chip. (3) Guide the metal/silicon composite slider to move correctly.

**Figure 3:**
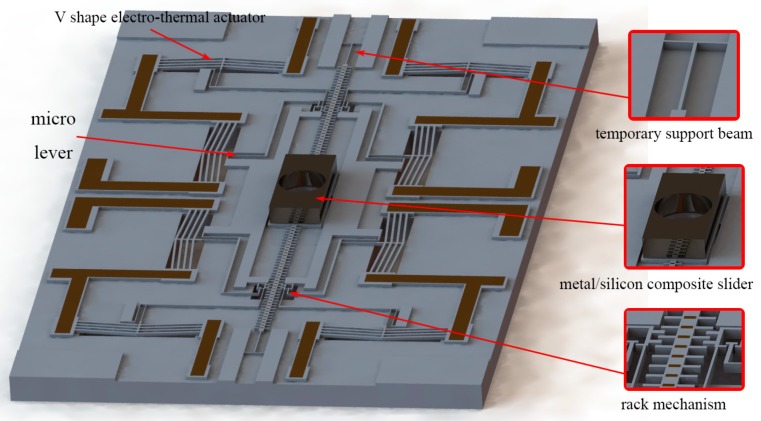
The structure of the actuation chip. The temporary support beam can prevent the composite slider from flowing away during the releasing process.

The actuation chip is the core component in the MEMS S&A device. An electro-thermal principle is applied to guarantee a sufficient output force and output displacement. Based on our previous research [18], the metal enhanced layer is added, and the MEMS stage in our early study has been modified into the MEMS S&A device. The metal/silicon composite slider is set in the center of the chip, and four pawl actuators are designed around the slider. This axisymmetric arrangement makes it easy to realize bidirectional actuation. In order to enhance the output performance, the structure of the backside cavity is necessitated. The composite slider is the biggest movable part of the device, and it is fixed to the substrate by the temporary support beam, as shown in Figure 3. For the purpose of reducing the jitter during the slider operation, a 5-µm wide gap between the movable structures should be guaranteed.

In summary, the MEMS S&A device that we present in this paper has complex structures and functions. A new fabrication flow needs to be developed and carefully arranged so as to realize the composite structure mobility, high accuracy, and a high-aspect ratio.

### 2.2. The Fabrication Flow

#### 2.2.1. The Cover Plate

**Figure 4:**
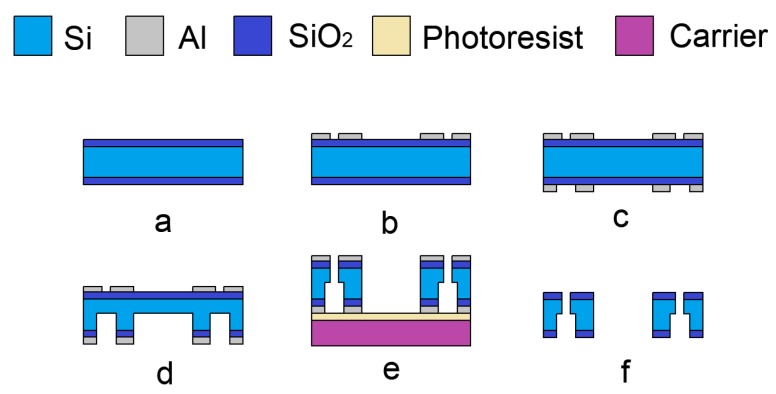
The fabrication flow of the cover plate. (**a**) The fabrication of insulation layer. (**b**) The fabrication of front side Al layer. (**c**) The fabrication of backside Al layer. (**d**) The fabrication of backside structure. (**e**) The fabrication of front side structure. (**f**) The cleaning.

The fabrication of the cover plate is carried out on 4-inch double-side polished wafers (KST, Fukui, Japan), p-type (1 0 0) with a resistivity of 1–20 Ω·cm and a thickness of 300 µm. The process flow is shown in Figure 4. 

Step A is the fabrication of the insulation layer. A 300-nm thick SiO_2_ is deposited on both sides of the wafer by plasma-enhanced-chemical-vapor-deposition (PECVD) (TRION ORION III, Arlington, TX, USA). The step introduced here is to guarantee the electrical insulation between the cover plate and the actuation chip. The parameters of the PECVD process are shown in Table 1.

Steps B and C are the fabrication of the double sides of the Al mask. A 200-nm thick Al is sputtered on both sides of the wafer, and then covered by the positive photoresist EPG 535 (Everlight Chemical, Taipei, Taiwan, China). After being exposed by ABM6 (ABM, San Jose, CA, USA) and developed by NaOH (0.5 wt%), the structure is transferred from the mask to the photosist. Then, the wafer is immersed into the aluminum etchant (80% H_3_PO_4_ + 5% HNO_3_ + 5% HAc + 10% H_2_O) at 60 °C for 30 s, and the pattern process can be accomplished.

Steps D and E, are the (inductively–coupled-plasma) ICP etching. The wafer will be etched by ICP on both sides, and the 200-µm deep cavity is formed on the backside firstly, as shown in Figure 4d. Because of the deep trench on the backside, gas leakage will occur during the etching process. In order to solve this problem, a 4-inch carrier is coated with photoresist EPG 535 (500 rpm, 5 s/1000 rpm, 20 s) and attached to the wafer. After being hard baked (90 °C; 10 min) together, those two wafers will bond temporarily. The photoresist can provide sufficient support and a thermal path during the ICP etching [19]. When this step is complete, the wafer is immersed in acetone for 30 min, and it will detach from the carrier automatically. 

Step F, the cleaning. The wafer is cleaned in a Piranha solution (H_2_SO_4_:H_2_O_2_ = 3:1, 120 °C, 5 min) [20,21], and the Al layer and the debris of photoresist will be removed simultaneously.

#### 2.2.2. The Actuation Chip

**Figure 5:**
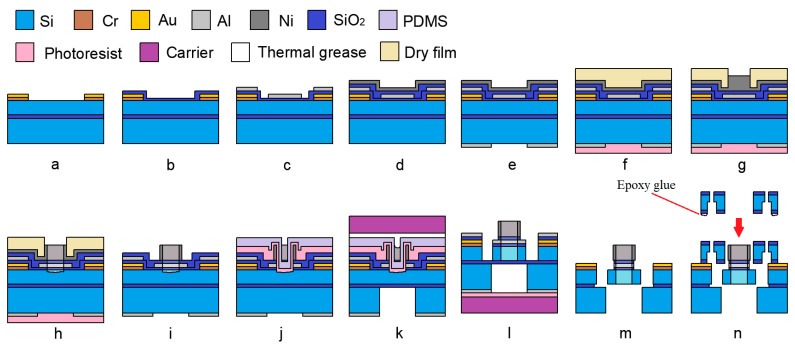
The fabrication process of the actuation chip. (**a**) The fabrication of gold pad. (**b**) The fabrication of adhesion layer. (**c**) The fabrication of front side Al layer. (**d**) The fabrication of insulation layer and Ni seed layer. (**e**) The fabrication of backside Al layer. (**f**) The fabrication of protection layer and dry film. (**g**) The fabrication of electroplating. (**h**) The laser process. (**i**) The removal of the dry film and the protection layer. (**j**) The fabrication of AZ 4620 and the PDMS protection layer. (**k**) The fabrication of backside structure. (**l**) The fabrication of front side structure. (**m**) The process of releasing. (**n**) The process of bonding.

Because of the movable structure in the actuation chip, the SOI (KST, Fukui, Japan) wafer is used here, and the parameters are listed in Table 2. The fabrication process is shown in Figure 5.

Step A is the fabrication of a gold pad. The gold pad is patterned on the device layer by lift-off. In order to have a good ohmic contact and stable pad surface for wire bonding, a 50-nm Cr layer and 200-nm Au layer are introduced.

Step B is the fabrication of the adhesion layer. In order to increase the adhesion of the substrate. A layer of 100-nm thick SiO_2_ is deposited on the wafer by PECVD.

Step C, the fabrication of front side Al layer. In order to avoid the structure distortion caused by the isotropic character of the Al etchant, the metal mask layer is formed by the process of lift-off. Hexamethyldisiloxane (HMDS) is covered on the wafer to guarantee the high quality of the exposed photoresist structure. After sputtering 50-nm thick Al, the wafer is immersed in acetone and then the structure of the Al mask can be formed.

Step D is the fabrication of the insulation layer and the Ni seed layer. A layer of SiO_2_ (100 nm) is deposited on the wafer by PECVD, and the function of insulation can be realized. Then, 200 nm Ni is sputtered on the wafer as the seed layer for electroplating.

Step E is the fabrication of the backside Al layer. Just like Step C above, a 200 nm thick Al mask is sputtered and patterned on the backside of the wafer.

Step F is the fabrication of the protection layer and dry film. A layer of photoresist EPG 535 is covered and hard baked on the backside of the wafer, which can protect the Al mask from damaging during the electroplating process. A 120-µm thick dry film SAF 2100 (DUPONT, Wilmington, DE, USA) is laminated three times (about 360 µm in thickness) on the front side of the wafer by the wafer mounter SWM-100 (HALTHAN, Shanghai, China). 

Step G is the fabrication of the electroplating. After being exposed, the 300-µm thick Ni plate is electroplated on the exposed area, and the parameters of the photolithography process and the electroplating process are shown in Table 3 and Table 4, respectively.

Step H is the laser process. Restrained by the low resolution of the dry film, the intricate structure with high aspect ratio is hard to be obtained. In order to refine the structure of the Ni plate, a femtosecond laser is introduced here. Different from normal laser processing, the pulse of the femtosecond laser is at the femtosecond level, and the action time of the pulse is much smaller than that of the electron lattice scattering. When the laser pulse is completed, the energy is too late to be transmitted to the lattice. As a consequence, this kind of cold processing has the feature of high accuracy and low damage to the surrounding material. By controlling the laser focus, a 3D structure with a high aspect ratio can be fabricated. After this step, a 310–320 µm deep blind hole is formed in the center of the Ni plate. The parameters of the fabrication process are shown in Table 5. It should be noted that the silicon structure beneath the removed Ni will be etched. Considering that the ultimate structure is a through-hole, the “structural damage” that occurred in this step is acceptable.

Step I is the removal of the dry film and the protection layer. A small amount of acetone is smeared around the wafer to avoid the Ni-structure damage caused by the swelling of the dry film. Then, the dry film and the wafer are gradually separated by the tweezers. The process of the oxygen plasma ashing (300 W; 30 min) is used here to remove the dry film residue, and the Ni seed layer will be exposed. Because of the protection of photoresist, the Al mask on the backside can stay intact in the Ni etchant (HCl:HNO_3_:H_2_O = 3:1:2, 30 °C, 2 min) [20,21]. After removing the Ni seed layer, the wafer is immersed in acetone and the photoresist protection layer can be dissolved.

Step J is the fabrication of AZ 4620 and polydimethylsiloxane (PDMS) protection layer. This is the preparation step for the ICP etching. A good thermal conductivity between the wafer and the carrier determines the high quality of etching. The grease, silicon oil, and paraffin are ideal materials for thermal conduction. Because of the thick Ni plate on the wafer, thermal grease is the suitable one. Considering that the thermal grease is hard to remove, the protection layer AZ 4620 and PDMS are introduced. AZ 4620 is sprayed evenly on the wafer. As the intermediate layer, AZ 4620 enables easy stripping of the wafer and the PDMS layer. A layer of 100 µm PDMS is coated on the wafer and hard baked at 80 °C for 15 min. Then, the wafer is smeared with a thermal grease and fitted with the carrier.

Step K the fabrication of backside structure. The handle layer of the SOI is etched to the buried layer by ICP, and a 400-µm deep cavity is formed. After the etching process, the wafer is detached from the carrier, and the thermal grease left on the wafer is removed by stripping off the PDMS layer. The photoresist layer is removed by acetone in order to expose the front side mask.

Step L, the fabrication of the front side structure. Just as the fabrication step in Figure 4e, the wafer is attached to the cleaned carrier again by the photoresist EPG 535. A layer of 200-nm thick SiO_2_ and 50-µm Si is etched by ICP successively. Then, the wafer is detached from the carrier by acetone.

Step M, the process of releasing. The Al mask on the wafer is removed by the Al etchant, and the buried layer is etched by buffered-oxide-etch (BOE; 40% NH_4_F:49% HF = 5:1, 25 °C, 40 min). Without the support of the buried layer, the suspended metal/silicon structure is fix to the substrate by the temporary support beam.

Step N is the process of bonding. The epoxy glue EPO 330 is dispensed on the assembled part of the cover plate and is aligned with the SOI wafer. With a proper temperature (110 °C) and pressure (0.1 MPa), the two wafers can be bonded together. The microscopy on the bonding machine can guarantee the alignment accuracy to ±5 µm. Except for the gold pads and the metal/silicon structures, the SOI wafer is well protected by the cover plate. Then, the bonded wafer is diced by the laser process. With the protection of the cover plate, the out-off-plane motion of the movable structure is well confined. Through the observation window, the temporary support beam is broken by the probe, and the final releasing step is complete.

## 3. Experimental Results and Discussion

### 3.1. The Fabrication Result of the Cover Plate

**Figure 6:**
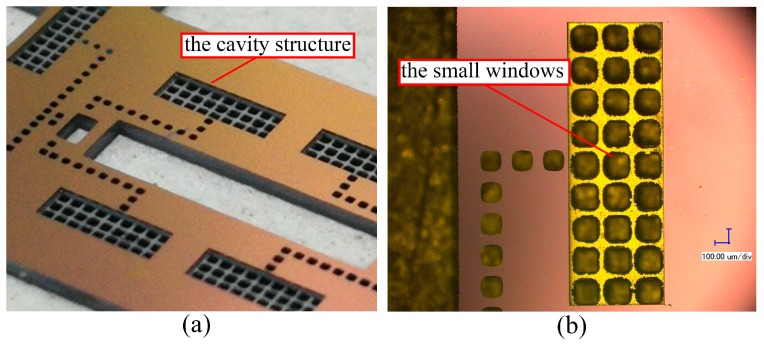
The fabrication result of the cover plate. (**a**) The structure of cavity. (**b**) The structure of the small windows.

The cover plate is double side-etched, and the fabrication result is shown in Figure 6. The 200-µm deep cavity structure formed on the back side is used to increase the thickness of the air gap between the actuation chip and the cover plate, as shown in Figure 6a. As the MEMS S&A device is driven by the electro-thermal principle, the thick air gap can increase the thermal resistance, which will enhance the thermal efficiency of the device drastically. The small windows (140 µm × 140 µm) on the front side are used to observe the motion of the electro-thermal actuator.

### 3.2. The Fabrication Result of the Actuation Chip

#### 3.2.1. The Process of High Precision Metal/Silicon Structure

In the MEMS S&A device, the 300-μm thick Ni plate needs to be fabricated on the movable silicon stage. What is more, a ϕ 0.85 mm × 0.3 mm cavity is designed in the center of the 1 mm × 3 mm × 0.3 mm Ni plate. The minimum width of the structure is 75 μm, while the thickness of the plate is 300 μm. As a result, the low resolution of the dry film, the structure cannot be formed. Therefore, the Ni plate is first electroplated on the wafer, and then the cavity is formed by the femtosecond laser. The process deviation is mainly from the positioning platform, and the measurement data is shown in Table 6, which is about 10 μm.

**Figure 7:**
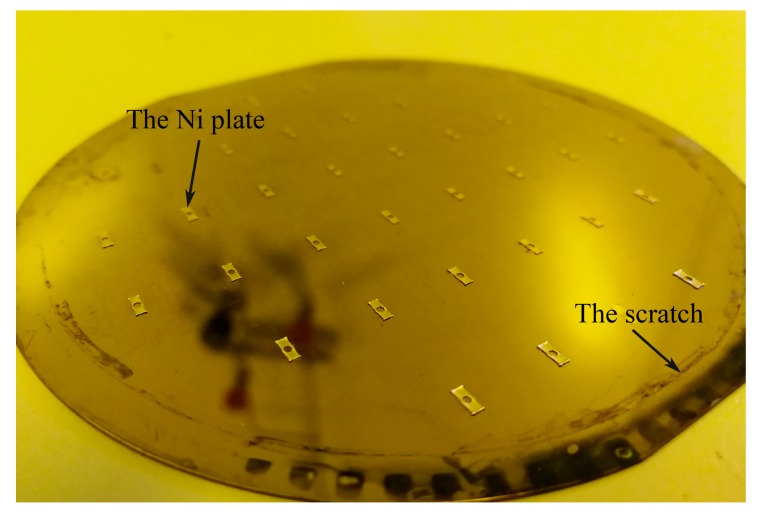
The fabrication result of electroplating and laser process.

The fabrication result of the metal/silicon structure is shown in Figure 7. The dry film SAF 2100 on the wafer is removed and cleaned. Then, the 300-μm Ni plate can be found fabricated firmly on the SOI wafer. The electroplating fixture may leave a small amount of scratches on the surface, but they will not affect the subsequent process. 

**Figure 8:**
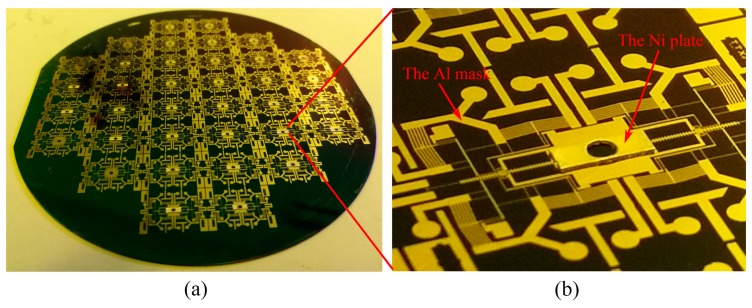
The fabrication result of metal/silicon structure. (**a**) The wafer after removing the Ni seed layer. (**b**) The image of the Ni plate (1 mm × 3 mm × 0.3 mm with the ϕ 0.85 mm × 0.3 mm cavity in the center).

The Ni seed layer is removed by the Ni etchant. Considering the protection of the SiO_2_ insulation layer, the Al mask can stay intact in the etchant, as shown in Figure 8. 

#### 3.2.2. The Process of ICP Etching

**Figure 9:**
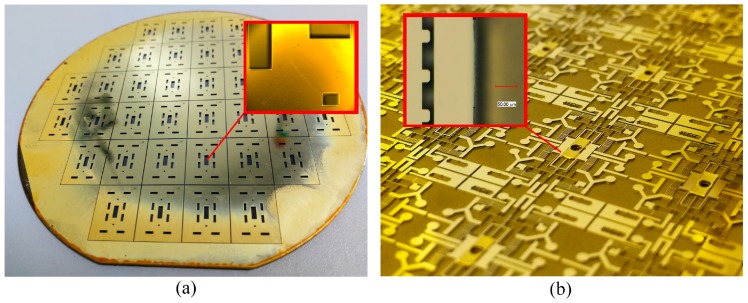
The fabrication result of (inductively–coupled-plasma) ICP etching. (**a**) The backside structure. (**b**) The front side structure.

**Figure 10:**
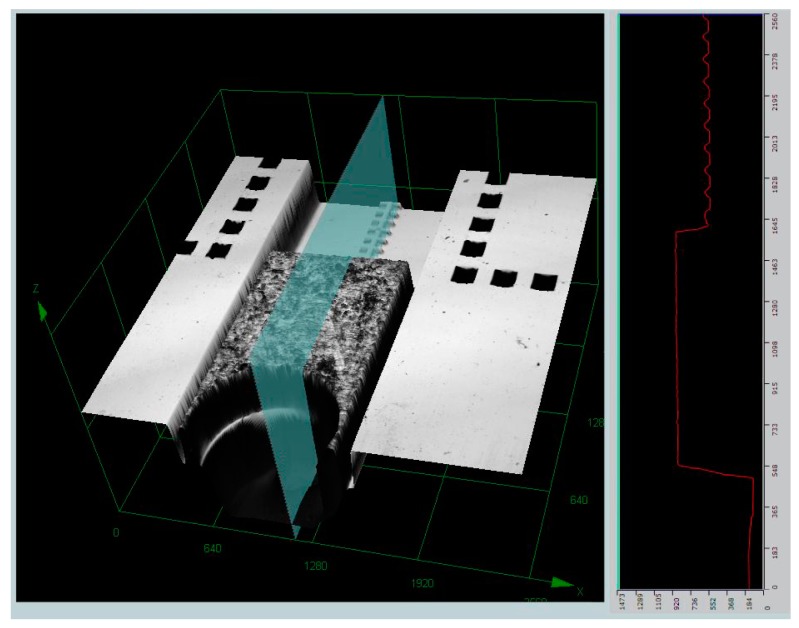
The inspection result of the Ni plate.

The ICP result is shown in Figure 9. The structure on the backside of the wafer is first etched, as shown in Figure 9a. Because of the buried layer in the SOI wafer, the etch depth 400-μm can be controlled precisely. Because of the protection layer of AZ 4620 and PDMS, the thermal conduction of the wafer will be affected during the etching process. Although thermal grease is used as the compensation, the verticality (about 85°) of the structure cannot be guaranteed. Fortunately, the structure on the backside does not require high precision, and the result is acceptable. The structure on the front side of the wafer is shown in Figure 9b, and gap of 50 μm in depth and 5 μm width can be obtained. The inspection result in the out-off-plane direction is obtained by the 3D profiler (OLYMPUS-OLS4000, Tokyo, Japan), as shown in Figure 10. There are 36 chips that can be formed in one wafer, and all of them are tested. The Ni plate thickness varies from 290 μm to 310 μm, and the average value is 303.6 μm.

#### 3.2.3. The Process of Releasing

**Figure 11:**
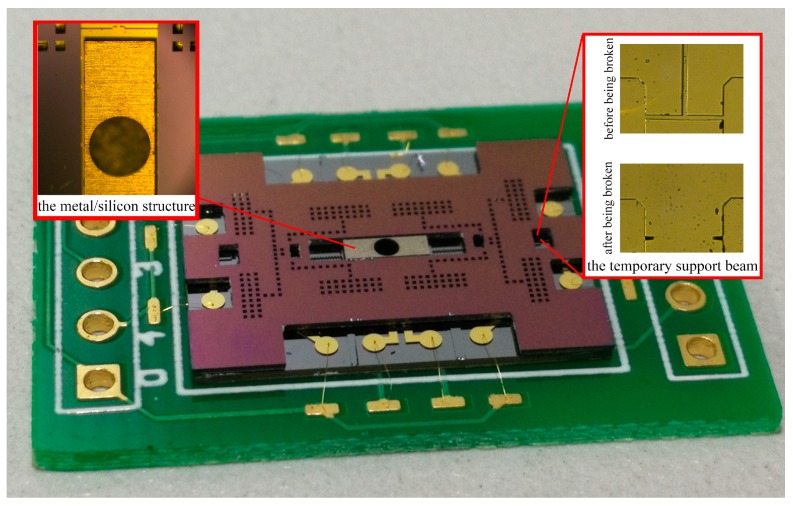
The ultimate structure of MEMS S&A device.

After releasing in BOE, the SOI wafer is bonded with the cover plate wafer using epoxy glue. Considering that the SOI surface quality can be hardly guaranteed after so many steps, the process of Au–Au bonding is not used here. All of the suspended structures are well protected and confined by the cover plate. In order to avoid the structure damage, the laser dicing process is applied, and the diced device is shown in Figure 11. The movable metal/silicon structure is fixed to the substrate by the temporary support beam. As a slender beam (1060 μm × 10 μm), it can be easily broken by the probe, while the metal/silicon structure will stay intact. Without the constrain of the temporary support beam, the metal/silicon structure can be fully released.

## 4. The Characterization of the MEMS S&A Device

### 4.1. The Test Platform

**Figure 12:**
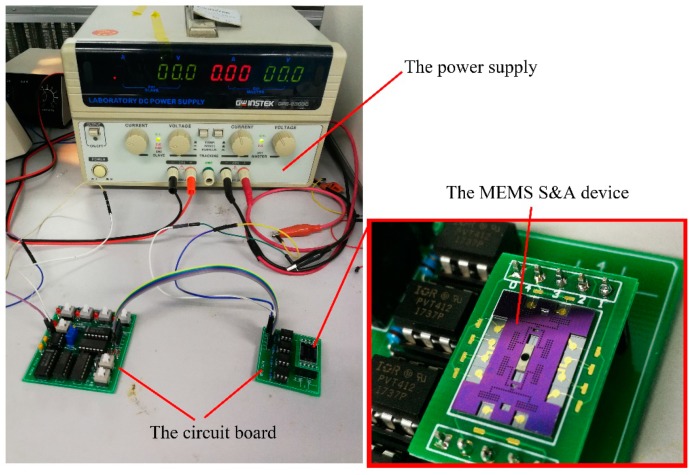
The test platform.

The movable performance is investigated to validate the design and fabrication of the MEMS S&A device. The test platform is composed of the power supply (GPC-6030D, Gwins, Taipei, Taiwan, China), the circuit board, and the MEMS S&A device, as shown in Figure 12. The working voltage of the circuit board is 5 V, while that of the MEMS S&A device is 11 V. In order to achieve the circuit board’s control over the MEMS S&A device, an optoelectronic switch (PVT 412, SiFotonics, Boston, MA, USA) is set here.

### 4.2. The Control Signal

**Figure 13:**
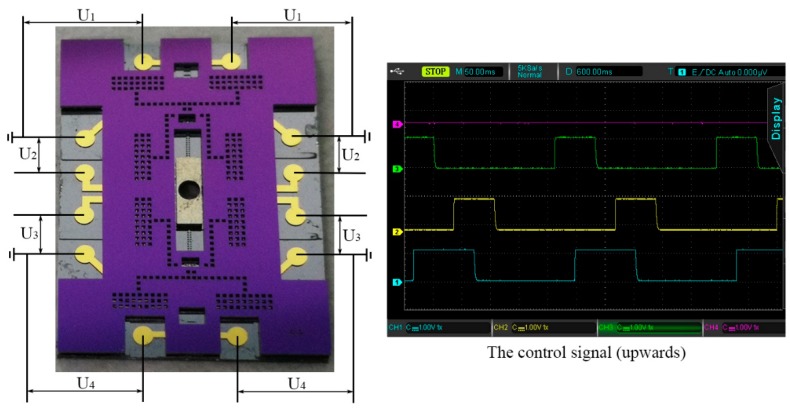
The control signal of the MEMS S&A device.

Four groups of control signals were applied in the specific sequence to achieve the actuation of the MEMS S&A device, as shown in Figure 13. As the response time of electro-thermal actuator is in milliseconds, the actuation period is set 300 ms in order to guarantee the sufficient response of the device. As a consequence, the device status conversion can be achieved in 3 s (10 steps), which indicates the fine time-delay function of the device. Moreover, by changing the resistance on the circuit board, the actuation period can be varied from 50 ms to 600 ms. Because of the symmetric arrangement of the structure, the actuation of the MEMS S&A device can be achieved in a bi-direction. The moving upward signals are similar to the moving downward signals, as shown in Figure 13. By swapping signal 1 and 4, and 2 and 3, the conversion in the driving direction can be realized.

### 4.3. The Moving Tests

The MEMS S&A device presented in this paper is driven by an electro-thermal principle, and the output force is about 15 mN under the 11 V control signal. As the metal/silicon structure is fully released, the whole slider will be in contact with the silicon substrate, and the impact of friction should be taken into consideration. In the MEMS scale, the frictional behaviors are affected by the material and surface, and the coefficient of friction can change widely [22,23,24,25]. Some of the published data are shown in Table 7. 

Considering that the working situation in our design is similar to that in the literature [24], the coefficient of friction was chosen to be 0.3. The weight of the metal/silicon structure is 66.5 μN (the silicon structure weights 2.8 μN and the metal structure weights 63.7 μN), and the friction force is 19.95 μN. Compared to the electro-thermal output force (15 mN), the friction force can be ignored. 

**Figure 14:**
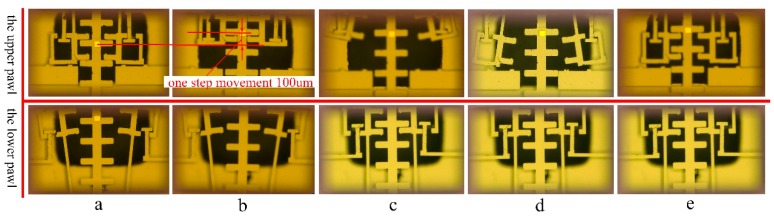
The sub-step movement of the MEMS S&A device. (**a**) The lower pawl disengagement. (**b**) The upper pawl pulling one sub-step. (**c**) The upper pawl disengagement and the lower pawl reengagement. (**d**) The upper pawl alignment. (**e**) The upper pawl reengagement. The MEMS S&A device accomplishes one step movement.

With the control signal mentioned in Section 4.2, every sub-step movement is captured, as shown in Figure 14a–e. The pulling displacement is measured as 100 μm, and the disengagement displacement is 45 μm. In Figure 14e, both of the upper pawl and the lower pawl have returned to the initial place, and the MEMS S&A device has accomplished one step movement. As the teeth on the pawl and slider will always mesh with each other, the movable slider can stay in the proper position during the sub-step actuation. If the wrong signal is applied, this special interlock mechanism can prevent the slider from moving away, which gives the device the function of signal recognition. 

**Figure 15:**
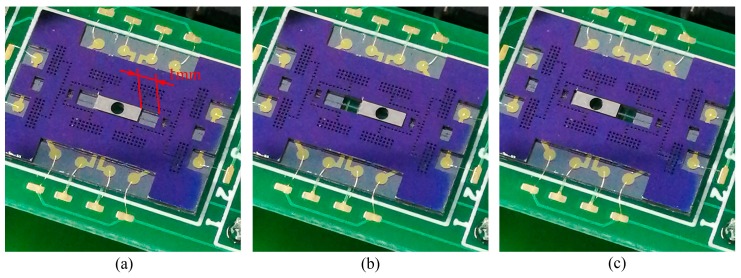
The moving tests of MEMS S&A device. (**a**) The armed position. (**b**) The safe position 1. (**c**) The safe position 2.

The MEMS S&A device is actuated at a full working range, and the result of the output displacement is shown in Figure 15. The device initial situation is shown in Figure 15a. The metal/silicon barrier lies in the center, and the whole device is in the armed position. Actuated by the proper control signal, the metal/silicon barrier can move in a bi-direction smoothly, which can realize the function of the status recovery (convert the status from safe to armed, or armed to safe), as shown in Figure 15b,c. The actuation displacement is 1 mm, while the diameter of the through hole in the center of the metal/silicon is 850 μm, which means that the acceleration barrel can be fully blocked during the safe position.

## 5. Conclusions

The fabrication process of a new kind of MEMS S&A device is presented in this paper. In order to overcome the fabrication difficulty of the metal/silicon composite structure, the hybrid fabrication process is introduced. This new method mainly consists of metal sputtering, electroplating, and ICP etching. As the resolution of the thick dry film is limited, the process of the femtosecond laser is applied to refine the structure, and the Ni plate (a block of 1 mm × 3 mm × 0.3 mm with a cavity of ϕ 0.85 mm × 0.3 mm in the center) is fabricated on the SOI wafer successfully. After the double sides are etched by ICP, the SOI wafer is immersed in a BOE etchant to remove the buried layer. The cover plate acts as the encapsulation, and is bonded with the SOI wafer by the epoxy glue. Then, the temporary support beam of the device is broken by the probe, and the suspended composite structure can be fully released. The hybrid process is the integration of the silicon-based process and the metal-based process, which can combine the advantages of both high precision and a high structure strength. The process proposed here is suitable for the application of weapon miniaturization. The actuation test of the MEMS S&A device is performed. Compared to the driven force (15 mN), the friction force (19.95 μN) can be ignored, and the whole device can realize 1 mm actuation in a bi-direction smoothly.

## Figures and Tables

**Table 1:** The PECVD process of SiO_2_.

Item	Paramter
Power	100 W
SiH_4_	200 sccm
N_2_O	140 sccm
N_2_	140 sccm
Temperature	350 °C
Time	60 s

**Table 2:** Parameters of silicon-on-insulator (SOI).

Item		Parameter
Diameter		100 mm
Orientation		(100)
Resistivity	Device layer	0.01–0.02 Ω·cm
	Handle layer	1–20 Ω·cm
Thickness	Device layer	50 μm
	Buried oxide layer	3 μm
	Handle layer	400 μm

**Table 3:** The photolithography process.

Material	Coating	Soft Bake	Expose	Post Bake	Develop	Hard Bake
Dry film SAF 2100	Laminated three times	65 °C 8 min	MA6 9.2 mW/cm^2^ 6 s	95 °C 55 s	NaCO_3_ 2 wt% 6 min	100 °C 2 min
PDMS	500 rpm, 9 s 2000 rpm, 30 s	80 °C 15 min	-	-	-	-
AZ 4620 (spray)	500 rpm, 30 s	85 °C 15 min	-	-	-	-

**Table 4:** The electroplating process of Ni.

Item	Parameter
Ni(NH_2_SO_3_)_2_·4H_2_O	550 g/L
NiCl_2_	10 g/L
H_3_BO_3_	35 g/L
Wetting agent	0.15 g/L
PH	3.9
Current	0.127 A
Temperature	50 °C
Time	20 h

**Table 5:** The process of the femtosecond laser.

Item	Parameter
Frequency	300 kHz
Power	1.8 W
Platform speed	800 mm/s
Time delay of opening	120 μs
Time delay of closing	70 μs
Time delay of ending	10 μs
Cycle	30

**Table 6:** The deviation of the laser process.

Object	Max	Min	Deviation
0.075 mm	0.0856 mm	0.0751 mm	0.0105 mm
ϕ 0.85 mm	0.8546 mm	0.8458 mm	0.0088 mm

**Table 7:** Coefficient of friction in micro scale.

Group	Materials	Contact Surface	Friction Coefficient
Lumbantobing [23]	Poly-Si/Poly-Si	Flat	0.29–0.86
Noguchi [24]	Si/Si	Flat	0.3
Wang [25]	SiN_x_ tip/Si	Flat	0.02–0.06
